# Clinical evaluation of the novel Capiox NX19 adult oxygenator–a multicenter study

**DOI:** 10.1177/02676591221078942

**Published:** 2022-03-29

**Authors:** Rik H J Hendrix, Gerdy Debeuckelaere, Karlien Degezelle, Lieven Lenaerts, Tom Verbelen, Patrick W Weerwind

**Affiliations:** 1 Department of Cardiothoraxic Surgery, 199236 Maastricht University Medical Centre, Maastricht, Netherlands; 2 Department of Extra-corporeal Circulation, 60202 University Hospital Antwerp, Edegem, Belgium; 3 Department of Perfusion Technology, 60182 University Hospitals Leuven, Leuven, Belgium; 4 Department of Cardiac Surgery, 60182 University Hospitals Leuven, Leuven, Belgium

**Keywords:** perfusion, oxygenator, Terumo Capiox NX19, clinical performance, oxygenator evaluation, cardiopulmonary bypass, microemboli, gas transfer, oxygen transfer, carbon dioxide transfer, heat exchanger performance

## Abstract

**Introduction:**

The novel Capiox NX19 adult oxygenator is, compared to its predecessors, improved with enhanced air removal technology, a polymer heat exchanger and smaller, innovative hollow fibers resulting in a surface area reduction and a lower priming volume. The aim of this study was to evaluate the NX19 oxygenator performance in a clinical setting.

**Methods:**

A prospective multicenter study was performed involving three large European university hospitals. The Capiox NX19 (*n* = 150) performance was assessed during adult cardiopulmonary bypass and involved gaseous microemboli handling and gas transfer efficiency. The heat exchanger performance was evaluated separately in vitro.

**Results:**

The heat exchanger performance factors were 0.80 ± 0.03 and 0.58 ± 0.04 at pump flow rates of 3 L/min and 6 L/min, respectively. After priming, residual post-oxygenator gaseous microemboli count and volume were decreased by 91% and 93.7%, respectively. The gas compartment pressure was 6.0 ± 2.5 mmHg, while the O_2_ transfer was 69 ± 30 mL/min/m^2^ and the CO_2_ transfer 73 ± 34 mL/min/m^2^. The O_2_ gradient was 44 ± 19 mmHg/LPM and the O_2_ diffusing capacity 0.38 ± 0.14 mL/min/mmHg. The shunt fraction was 0.19 ± 0.13, whereas oxygenator resistance and shear stress were 10.5 ± 3.7 mmHg/LPM and 5.1 ± 3.1 dyn/cm^2^, respectively.

**Conclusion:**

This multicenter study displayed good clinical safety and performance of the NX19 oxygenator.

## Introduction

The oxygenator plays a pivotal role in clinical cardiopulmonary bypass (CPB), as poor oxygen delivery can lead to significant patient morbidity and mortality.^1,2^ Although CPB has become widespread, oxygenator design is still advancing in order to make them more efficient, hemocompatible, and easier to use. Recently, a controlled launch of the Capiox NX19 oxygenator (Terumo Corporation, Tokyo, Japan) was initiated. This full-size adult oxygenator underwent several changes compared to its predecessors like the Capiox FX series. The heat exchanger has been altered from stainless steel to a thermoplastic polymer (polypropylene terephthalate) to improve heat exchange performance, and is adapted with proprietary pre-heat exchanger air removal technology to enable easier air removal during priming. Perhaps the most important change is the incorporation of smaller microporous polypropylene gas exchange fibers, reducing priming volume, surface-contact area, and transmembrane pressure drop.

This multicenter study was set up in order to perform a clinical evaluation to assess gaseous microemboli (GME) handling, gas transfer capacity and heat exchanger efficiency of the new Capiox NX19 oxygenator.

## Methods

### Centers and patients

Participating centers included the University Hospital Antwerp (Antwerp, Belgium), the University Hospital Leuven (Leuven, Belgium), and the Maastricht University Medical Center (Maastricht, the Netherlands).

Clinical evaluation of the Capiox NX19 was performed using 50 oxygenators during adult CPB surgery in each center (total *N* = 150). Adult patients scheduled for elective surgical revascularization of coronary arteries (CABG) and/or aortic valve replacement/repair (AVR/AVP) with CPB were included. Exclusion criteria comprised emergency surgery; renal disease defined as laboratory tests indicating an eGFR <50 mL/min and/or values of two or more times the normal values of urea (≥50 U/L) and creatinin (≥170 μmol/L); liver diseases defined as laboratory tests indicating values of two or more times the normal values (ASAT ≥50 U/L, ALAT ≥60 U/L, LD ≥600 U/L and gamma-GT ≥90 U/L); and participation in an investigational drug trial within the preceding 30 days. Data acquisition and data analyses were performed anonymously and included only routine care during CPB without the need for any intervention. In accordance with the Dutch law for approving medical research, the local medical review ethics committee approved the study as non-medical research involving human subjects (METC-number 2018-0915). Therefore, the necessity of informed consent was waived at Maastricht University Medical Center. According to the Belgian law of 7 May 2004 on experiments on the human person, the UZ Leuven ethical committee approved this study (S62853). All Belgian patients included signed an informed consent.

### Heat exchanger performance test

The heat exchanger performance of the Capiox NX19 oxygenator was evaluated in a set-up similar to the guidance for cardiopulmonary bypass oxygenators 510 (k) specifications.^3^ The performance of the NX19 heat exchanger was assessed in vitro using fixed venous temperatures (25, 30, 32, 34 and 36°C) delivered via a second oxygenator (Quadrox-i adult, Getinge, Gothenburg, Sweden). A Jostra HCU30 (Getinge) was connected to the Quadrox-i and set to a temperature required to reach the desired venous temperature, whereas another Jostra HCU30 was connected to the NX19 and set to 40°C. Jostra HCU30 water flows were approximately 8 L/min. The CPB system was primed with water and measurements were performed at pump flow rates of 3 and 6 L/min. When temperatures had stabilized, all temperatures (pre-NX19, post-NX19 and HCU35 water temperature) were registered every 5 minutes for a duration of 30 min. These data were used to calculate the NX19 heat exchanger efficiency, expressed as a heat exchanger performance factor. All formulae can be found in the online supplementary material.

### Clinical evaluation

The Capiox NX19 oxygenator was combined with a custom tubing set (Terumo Corporation) in each center. A CDI500 blood parameter monitoring system (Terumo Corporation) was incorporated to continuously measure both arterial and venous blood gas parameters. Priming was performed according to local institutional protocol and instructions for use of the NX19 oxygenator. This included circuit carbon dioxide flush prior to priming in Leuven and Maastricht, but not in Antwerp. After priming, a bubble count was carried out using the GAMPT BCC300 or BCC200 (GAMPT mbH, Merseburg, Germany). The amount of GME <250 μm was measured before and after the oxygenator during 1 minute at a continuous pump flow rate of 5 L/min. The volume in the cardiotomy reservoir was kept above the minimum operating level (150 mL) during the bubble count.

Cardiopulmonary bypass was performed according to the local hospital protocol, including institutional temperature management. Temperature regulations were defined as: normothermia (>35°C); mild hypothermia (35-32°C); moderate hypothermia (32-28°C); and severe hypothermia (28-18°C). Inline blood gas parameters measured during cardiac arrest using the CDI500 were used for analysis of oxygen transfer. Oxygenator exhaust CO_2_ measured via capnography was used for calculation of carbon dioxide transfer. Additionally, hemoglobin and hematocrit were determined by blood gas analysis just before the start of CPB and right after weaning from CPB. Platelet count and fibrinogen were determined the day before surgery and shortly after surgery. Pre- and post-oxygenator line pressures were measured to evaluate the effect of the smaller blood flow path on oxygenator resistance, and pressures in the gas compartment were measured to assess the effect of the smaller diameter gas exchange fibers. Perfusion data were digitally recorded using the institutional patient data management systems, all recording at frequencies of 3–4 records per minute. A case report form was used to collect anonymous patient demographics, CPB and aortic cross clamp times, temperature management and blood product usage.

Oxygenator performance was analyzed using the following parameters: O_2_ transfer; CO_2_ transfer; O_2_ gradient; O_2_ diffusing capacity; O_2_ transfer slope; shunt fraction; oxygenator resistance and shear stress. An overview of all used formulae can be found in the online supplementary material.

Descriptive statistics to determine patient demographics and operative characteristics were performed using SPSS 25 (IBM Corp. Chicago, IL, USA). Values are expressed as mean ± SD unless mentioned otherwise.

## Results

### Heat exchanger performance

**Figure 1.**
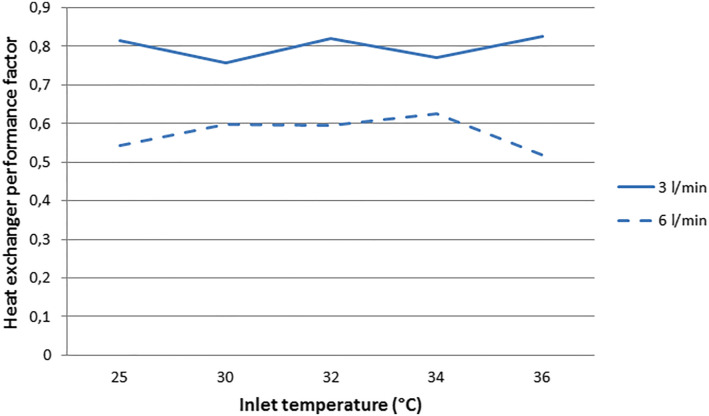
Heat exchanger performance factor at different inlet temperatures and pump flows.

Figure 1 depicts the in vitro assessed heat exchanger performance at different inlet temperatures. At a pump flow of 3 L/min, the heat exchanger performance factor was 0.80 ± 0.03, which decreased to 0.58 ± 0.04 at 6 L/min.

### Clinical evaluation

All patients were included between October 2019 and February 2021. Demographics and operative characteristics are shown in Table 1.

**Table 1.** Patient demographics and operative characteristics.

Male gender (*n* (%))	121 (81%)
Age (years)	66 ± 11
Height (cm)	173 ± 10
Weight (kg)	85 ± 17
BMI	28 ± 5
Operation type (*n* (%))	
CABG	73 (49%)
AVR	57 (38%)
CABG + AVR	20 (13%)
Temperature management (*n* (%))	
Normothermia	84 (56%)
Mild hypothermia	50 (33%)
Moderate hypothermia	16 (11%)
Deep hypothermia	0 (0%)
Cardiopulmonary bypass time (min)	93 ± 39
Aortic cross clamp time (min)	67 ± 32
Blood products used (*n* (%))	
None	126 (84%)
PRC	22 (15%)
Platelets	4 (3%)
FFP	2 (1%)

BMI: body mass index; CABG: coronary artery bypass grafting; AVR: aortic valve replacement; PRC: packed red blood cells; FFP: fresh frozen plasma.

During the priming procedures no particularities were noted. The main component of the priming solution was different in all three hospitals, i.e. Gelofusin 4% in Maastricht, Volulyte 6% in Antwerp, and Isogelo in Leuven. After priming, the average removal of GME volume by the NX19 was 93.7%, the average removal in GME count 91.0%. Pre- and post-oxygenator values and removal percentages per center are listed in Table 2.

**Table 2.** Gaseous microemboli removal efficiency of the NX19 oxygenator.

	Microemboli count (<250 μm)			Microemboli volume		
	Pre-oxygenator (*n*)	Post-oxygenator (*n*)	Removal (%)	Pre-oxygenator (μm)	Post-oxygenator (μm)	Removal (%)
Maastricht	52.48	1.42	97.3	0.0637	0.0001	99.2
Antwerp	43.84	3.08	92.9	0.0477	0.0003	95.3
Leuven	94.12	11.78	84.2	0.0096	0.0002	88.6

**Figure 2.**
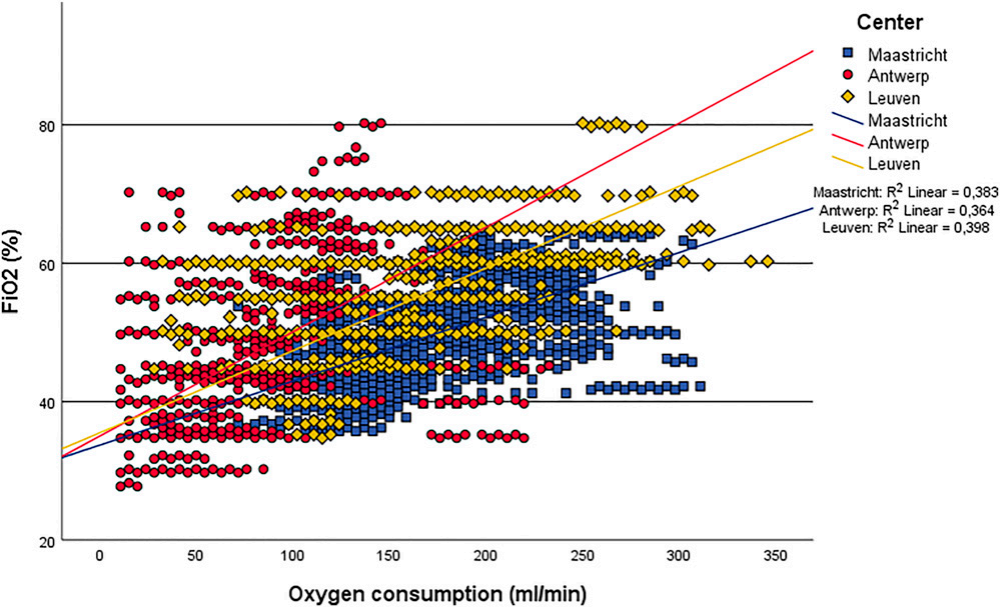
O2 transfer slope.

All parameters regarding oxygenator efficiency were assessed using data collected during the aortic occlusion period and are shown in Table 3. The O_2_ transfer slope is depicted in Figure 2.

**Table 3.** NX19 oxygenator efficiency parameters.

O_2_ transfer, ml/min/m^2^ (*N* = 150)	69 ± 30
CO_2_ transfer, ml/min/m^2^ (*N* = 150)	73 ± 34
O_2_ gradient, mmHg/LPM (*N* = 150)	44 ± 19
O_2_ diffusing capacity, ml/min/mmHg (*N* = 150)	0.38 ± 0.14
Shunt fraction, Q_s_/Q_t_ (*N* = 150)	0.19 ± 0.13
Oxygenator resistance, mmHg/LPM (*N* = 150)	10.5 ± 3.7
Shear stress, dynes/cm^2^ (*N* = 150)	5.1 ± 3.1
Gas pressure, mmHg (*N* = 100)	6.0 ± 2.5

Packed red blood cell (RBC) transfusions were given in 21 patients (14%) and platelet transfusions to three patients (2%). In the patients not receiving RBC transfusions, hemoglobin content decreased 3.2 gr/dl with a corresponding decrease in hematocrit of 10% (Table 4). In patients not receiving platelet transfusions the platelet count decreased with 98 10^9^/l. The decline in fibrinogen level during CPB was 1.1 gr/l.

**Table 4.** Pre- and postoperative blood component values.

	Preoperative value	Postoperative value
Hemoglobin, gr/dl (*N* = 129)	13.4 ± 1.4	10.2 ± 1.4
Hematocrit, % (*N* = 129)	39 ± 5	29 ± 4
Platelet count, x10^9^/l (*N* = 147)	237 ± 64	139 ± 46
Fibrinogen, gr/l (*N* = 150)	3.3 ± 1.0	2.1 ± 0.7

## Discussion

This multicenter study reports a clinical evaluation of the recently launched NX19 oxygenator, assessing heat exchanger performance, GME handling and gas transfer efficiency.

The mean heat exchanger performance factor of 0.80 ± 0.03 found at a pump flow rate of 3 L/min, is in line with the one reported in the NX19 instructions for use (IFU; 0.83 at 3 L/min)^4^ and higher than the performance factor reported in the FX25 IFU (0.74 at 3 l/min).^5^ At 6 L/min the mean performance factor was 0.58 ± 0.04, which is lower than reported in the NX19 IFU (0.68 at 6 L/min), but equal to the performance factor reported in the FX25 IFU (0.58 at 6 L/min).

Besides the effect on heat transfer, the change from a stainless steel to a polymer heat exchanger has also improved biocompatibility, reducing platelet contact activation and thrombus susceptibility.^6^ Although analysis of platelet activation was beyond the scope of this evaluation, platelet count decreased by 29%, which is comparable with the 20–30% decrease seen in other studies.^7,8^ Postoperative fibrinogen levels were similar to the levels measured by Erdoes et al.,^9^ who showed recovery to normal within 13 h postoperatively. They were also well above the trigger for fibrinogen supplementation to prevent serious bleeding (1.15 g/l) found by Ranucci et al.^10^ This shows no excessive platelet or fibrinogen consumption by the NX19 oxygenator.

Gaseous microemboli handling was assessed after priming the CPB system, which was performed according to local institutional protocol and instructions for use of the NX19 oxygenator. The NX19 showed good processing of GME, decreasing the mean microemboli count with 91.0% and preventing the passage of 93.7% of the GME volume. With regard to GME count, the removal efficiency of the NX19 is similar to the 89.6% decrease in GME count of the Capiox FX25 found by Johagen et al.^11^ Although the NX19’s efficiency to handle GME is lower than the 99.1% removal found in the FX25 by Johagen et al., it is similar to the 95.03% removal by the Capiox FX25 measured by Stehouwer et al.^12^ There were differences in microemboli count and volume between the three participating centers, and in contrast to known literature, the center not using CO_2_ flush had the lowest pre-oxygenator microemboli count.^13,14^ This result, and other differences in microemboli count and volume between centers, can be attributed to the use of different custom packs, different priming fluids, as well as differences in priming technique. As the microembolic count and volume were small and no particularities occurred during any of the 150 priming rounds, the NX19 oxygenator showed to be effective in removing GME, despite the technical differences between centers.

Patients not receiving RBC transfusions had a mean postoperative hemoglobin value of 10.2 ± 1.4 gr/dl, well above the transfusion level in all three centers. The effect of the 75 mL reduction in priming volume of the NX19 (compared to the FX25) on hemoglobin concentration and PRC transfusions was not specifically addressed in this evaluation as all three centers use different priming volumes and transfusion thresholds. The effect of the reduction in oxygenator priming volume could however be important in centers using minimized CPB circuits and in patients with small circulating blood volumes and/or low preoperative hemoglobin levels.

Because of the smaller hollow fibers and their more compact arrangement, the width of the blood flow path through the oxygenator decreased. This did not instigate an increase in oxygenator resistance, which with a mean of 10.5 ± 3.7 mmHg/LPM, is on the low end compared to other contemporary oxygenators. Stanzel and Henderson^8^ calculated similar resistances in the Capiox FX25 (10 ± 0.25 mmHg/LPM) and in the Quadrox-i (8.4 ± 0.14 mmHg/LPM). They found higher resistances in the Fusion (16 ± 0.23 mmHg/LPM), the Inspire 8F (27 ± 0.67 mmHg/LPM) and the Inspire 6F (30 ± 0.82 mmHg/LPM). The mean shear stress in the NX19 (5.1 ± 3.1 dyn/cm^2^) did slightly increase compared to the FX25 (2.7 ± 0.6 dyn/cm^2^) and is higher than the Quadrox-i (2.1 ± 0.4 dyn/cm^2^).^15^ Nevertheless, the shear stress rates of the NX19 are still far below those leading t blood component damage.^16^ The smaller inner diameter of the hollow fibers could have caused elevated pressures in the gas compartment, with the risk of air leakage into the blood compartment. The mean gas compartment pressure of 6 ± 2.5 mmHg however, is very low compared to pressures on the blood side, minimizing the risk of air leakage.

The mean NX19 O_2_ transfer (69 ± 30 mL/min/m^2^) revealed to be higher than values calculated for the Capiox FX25 (44 ± 14 mL/min/m^2^).^15^ Carbon dioxide transfer rates showed an improvement as well with a mean of 73 ± 34 mL/min/m^2^ for the NX19 compared to 26 ± 14 mL/min/m^2^ for the Capiox FX25,^15^ illuminating an overall superior gas transfer efficiency of the NX19 oxygenator. This efficiency was confirmed by the other calculated O_2_ transfer parameters. The mean O_2_ gradient (44 ± 19 mmHg/LPM) was similar to values found for Capiox FX25 (45 mmHg/LPM), Inspire 8F (46 mmHg/LPM) and Quadrox-i (48 mmHg/LPM) at a blood flow rate of six LPM.^17^ At a flow rate of four LPM however, Hendrix et al. calculated higher mean O_2_ gradients of 51 mmHg/LPM for Capiox FX25, 58 mmHg/LPM for Inspire 8F and 53 mmHg/LPM for Quadrox-i. The mean O_2_ diffusing capacity of the NX19 (0.38 ± 0.14 mL/min/mmHg) was lower than that calculated for the Quadrox (0.48 ± 0.09 mL/min/mmHg) in a study by Segers.^18^ Both results indicate good O_2_ transfer performance of the NX19 oxygenator. The oxygen transfer slope (Figure 2) shows that challenging O_2_ consumption rates of 200–300 mL/min can be easily met with normal FiO_2_ levels of 40–60%. The differences seen in O_2_ consumption between the centers can be attributed to differences in temperature management. Antwerp, that showed the lowest O_2_ consumption, did 34 cases using mild hypothermia and 14 cases with moderate hypothermia. Maastricht had the highest O_2_ consumption, but used mild hypothermia only twice and performed all other cases with normothermia. Leuven is in between in both O_2_ consumption and temperature management (14 mild and two moderate hypothermia cases). Another important factor influencing O_2_ consumption, the anesthetic technique, has not been evaluated in this study.

The mean shunt fraction of the NX19 was 0.19 ± 0.13, whereas the mean shunt fraction of the FX25 was 0.32 ± 0.1,^15^ indicating that in the NX19 oxygenator a considerably smaller part of the blood travels through the oxygenator without effective gas transfer.

In conclusion, the change from a stainless steel to a polymer heat exchanger improved the heat exchanger performance of the NX19 compared to its predecessors. The incorporation of smaller gas exchange fibers not only decreased priming volume, but enhanced gas transfer efficiency as well, with little effect on gas inlet pressure or oxygenator shear stress.

## Supplemental Material

sj-pdf-1-prf-10.1177_02676591221078942 – Supplemental material for Clinical evaluation of the novel Capiox NX19 adult oxygenator–a multicenter study Click here for additional data file. Supplemental material, sj-pdf-1-prf-10.1177_02676591221078942 for Clinical evaluation of the novel Capiox NX19 adult oxygenator–a multicenter study by Rik H J Hendrix, Gerdy Debeuckelaere, Karlien Degezelle, Lieven Lenaerts, Tom Verbelen and Patrick W Weerwind in Perfusion
