# Supplementary material for: Clinical evaluation of the novel Capiox NX19 adult oxygenator–a multicenter study
Source: Perfusion. 2022 Mar 29;38(4):734–9. doi: 10.1177/02676591221078942 (PMC10102914; doi:10.1177/02676591221078942)
Supplement: sj-pdf-1-prf-10.1177_02676591221078942 – Supplemental material for Clinical evaluation of the novel Capiox NX19 adult oxygenator–a multicenter study [file sj-pdf-1-prf-10.1177_02676591221078942.pdf]

Supplementary material accompanying the manuscript entitled

"Clinical evaluation of the novel Capiox NX19 adult oxygenator - a multicenter study"

Rik H.J. Hendrix, Gerdy Debeuckelaere, Karlien Degezelle, Lieven Lenaerts, Tom Verbelen, Patrick W. Weerwind

All parameters calculated to evaluate the Terumo Capiox NX19 oxygenator heat exchanger efficacy and gas transfer efficiency, with corresponding formulae:

1) *Heat exchanger efficiency*. The heat exchanger efficiency was evaluated in an experimental set-up and expressed as the heat exchanger performance factor (R) [1]:

$$R = \frac{T_{post} - T_{pre}}{T_{water} - T_{pre}}$$

2) *Oxygen transfer*. Oxygen transfer was calculated as the amount of oxygen transferred to the blood per minute per square meter of membrane surface area [1].

$$O_2 \text{ transfer } (ml/min/m^2) = \frac{(CaO_2 - CvO_2) \times 10 \times Q_b}{MSA}$$

Where 10 is a conversion factor (deciliter to liter), Q_b is blood flow (l/min), MSA is the membrane surface area (m^2) and CaO_2 and CvO_2 are arterial and venous O_2 content respectively (ml/dl), calculated as follows [1]:

$$CaO_2 \text{ (ml/dl)} = (Hb \times 1.34 \times SaO_2) + PaO_2 \times 0.00314$$

CvO_2 (ml/dl) = (Hb × 1.34 × SvO_2) + PvO_2 × 0.00314

Where Hb is the hemoglobin content (gr/dl), SaO_2 and SvO_2 are the arterial and venous O_2 saturation (fraction), respectively, and PaO_2 and PvO_2 are the arterial and venous O_2 partial pressures (mmHg), respectively. 1.34 is a constant (ml O_2/gram Hb), as is 0.00314 (ml O_2/dl).

3) *CO_2 transfer.* The amount of carbon dioxide transferred by the oxygenator per minute per square meter membrane surface area was calculated using the following equation [2]:

$$CO_2 \text{ transfer (ml/min/m}^2\text{)} = \left(\left(Q_g \times 1{,}000 \times {CO_2}_{out} / AP \right) - (1 - (FiO_2 - 0.21)) \times Q_g \times 0.0003 \times 1{,}000 \right) / MSA$$

Where Q_g is sweep gas flow (l/min), 1,000 is a conversion factor (l/min to ml/min), ${CO_2}_{out}$ is the exhaust CO_2 (kPa) and AP is the atmospheric pressure (kPa). FiO_2 is the fraction of O_2 in the sweep gas (decimal form) and 0.21 and 0.0003 are constants (fractions of O_2 and CO_2 in room air, respectively).

4) *O_2 gradient*. The gradient between the partial O_2 pressure in the gas phase and the resultant arterial O_2 pressure shows the oxygenators ability to diffuse O_2. When shown as a function of blood flow, the influence of reduced transit time becomes visible [3].

$$O_2 \text{ gradient (mmHg/LPM)} = {}^{PgO_2 - PaO_2}\!/\!_{Q_b}$$

Where PgO_2 is the O_2 partial pressure in the gas phase (mmHg).

5) *O_2 diffusing capacity*. The pressure differential needed to transfer one milliliter of oxygen per minute was calculated as follows [3]:

$$O_2 \text{ diffusing capacity (ml/min/mmHg)} = {}^{O_2 \text{ transfer}}\!/\!_{PgO_2 - PvO_2}$$

6) *O_2 transfer slope*. This slope can be calculated by plotting the O_2 consumption against the corresponding FiO_2 in a scatterplot. The slope of a best-fit line reflects the change in FiO_2 needed per ml increase in O_2 consumption. O_2 consumption was calculated as follows [4]:

$$O_2 \text{ consumption (ml/min)} = (CaO_2 - CvO_2) \times Q_b$$

7) *Shunt fraction*. The fraction of the venous blood entering the oxygenator that exits without being oxygenated was calculated using the following equation [3]:

$$\text{Shunt fraction} = {}^{CiO_2 - CaO_2}\!/\!_{CiO_2 - CvO_2}$$

Where CiO_2 is the ideal oxygen content (ml/dl), calculated as follows [5]:

CiO_2 (ml/dl) = (Hb × 1.34 × 1) + (AP × FiO_2 × 0.00314)

Where the 1 is the decimal form for 100% saturated blood.

8) *Oxygenator resistance*. The transmembrane pressure drop corrected for blood flow [6]:

$$\text{Resistance (mmHg/LPM)} = {P_{pre} - P_{post}}/{Q_b}$$

Where P_{pre} and P_{post} are pre- and post-oxygenator pressures (mmHg) respectively.

9) *Shear stress (τ).* Oxygenator shear stress was calculated as follows [7]:

$$\tau\ (\text{dynes/cm}^2) = \sqrt{{Q_b \times \Delta p \times \mu}/{V}}$$

Where Δp is the transmembrane pressure drop (mmHg), μ is the dynamic blood viscosity (Pa*sec) and V is the volume of the oxygenator (L). Blood viscosity was calculated based on the hematocrit value [8]:

μ (Pa*sec) = exp(0.02345 × Ht + tt) × 0.001

With Ht being hematocrit (%) and tt a temperature transmitter to account for the effect of temperature [8]:

tt (°C) = exp (-0.0487 × T + 0.9213)

Where T is the arterial blood temperature (°C).

References:

1. Food and Drug Administration. Guidance for cardiopulmonary bypass oxygenators 510 (k) submissions; final guidance for industry and FDA staff, https://www.fda.gov/medical-devices/guidance-documents-medical-devices-and-radiation-emitting-products/cardiopulmonary-bypass-oxygenators-510k-submissions-final-guidance-industry-and-fda-staff (accessed 02-02-2021).
2. Hendrix RHJ, Ganushchak YM and Weerwind PW. Contemporary oxygenator design: shear stress-related oxygen and carbon dioxide transfer. *Artif Organs* 2018; 42: 611-619.
3. Jegger D, Tevaearai HT, Mallabiabarrena I, et al. Comparing oxygen transfer performance between three membrane oxygenators: effect of temperature changes during cardiopulmonary bypass. *Artif Organs* 2007; 31: 290-300.
4. Segers PA, Heida JF, de Vries I, et al. Clinical evaluation of nine hollow-fibre membrane oxygenators. *Perfusion* 2001; 16: 95-106.
5. Visser C and de Jong DS. Clinical evaluation of six hollow-fibre membrane oxygenators. *Perfusion* 1997; 12: 357-368.
6. Hjarpe AK, Jeppsson A, Lannemyr L, et al. Risk factors and treatment of oxygenator high-pressure excursions during cardiopulmonary bypass. *Perfusion* 2021: 2676591211043700. DOI: 10.1177/02676591211043700.
7. De Somer F. Does contemporary oxygenator design influence haemolysis? *Perfusion* 2013; 28: 280-285.
8. Riley JB and Zaidan JR. The immediate hemodynamic and metabolic effects of bolus injection of pharmacologic agents during cardiopulmonary bypass. *J Extra-Corpor Technol* 1983; 15: 71-77.
